# Hydrogen-Bonded Conjugated Materials and Their Application in Organic Field-Effect Transistors

**DOI:** 10.3389/fchem.2021.723718

**Published:** 2021-08-24

**Authors:** Xin Shi, Weiwei Bao

**Affiliations:** National and Local Joint Engineering Laboratory for Slag Comprehensive Utilization and Environmental Technology, School of Materials Science and Engineering, Shaanxi University of Technology (SNUT), Hanzhong, China

**Keywords:** hydrogen bonding, conjugated materials, small molecules, polymers, organic field-effect transistors

## Abstract

Recent research on organic semiconductors has revealed that the composition of the constituent organic material, as well as the subtle changes in its structure (the stacking order of molecules), can noticeably affect its bulk properties. One of the reasons for this is that the charge transport in conjugated materials is strongly affected by their structure. Further, the charge mobility increases significantly when the conjugated materials exhibit self-assembly, resulting in the formation of ordered structures. However, well-organized nanostructures are difficult to obtain using classical solution processing methods, owing to their disordered state. A simple strategy for obtaining well-ordered material films involves synthesizing new conjugated materials that can self-organize. Introducing hydrogen bonding in the materials to yield hydrogen-bonded material superstructures can be a suitable method to fulfill these critical requirements. The formed hydrogen bonds will facilitate the assembly of the molecules into a highly ordered structure and bridge the distance between the adjacent molecules, thus enhancing the intermolecular charge transfer. In this minireview, hydrogen-bonded small molecules and polymers as well as the relationship between their chemical structures and performances in organic field-effect transistors are discussed.

## Introduction

Hydrogen bonds have been receiving increasing attention by scientists since 1989 when Peter Atkins stated that “hydrogen bonding, a noncovalent interaction in structural organic chemistry, is a link formed by a hydrogen atom lying between two strongly electronegative atoms” (Atkins, 1989). A hydrogen atom can be shared between a hydrogen-bond donor and a hydrogen-bond acceptor, i.e., a molecular with electron lone pairs. Thus, hydrogen bonds exist ubiquitously, including in biological systems, dyes and pigments, ionic conductors, and organic semiconductors (Cooksey, 2001; Meot-Ner, 2005; Glowacki et al., 2013). Furthermore, hydrogen bond, as a kind of directional intermolecular interaction, can affect the configuration and optical physical properties of the molecules forming hydrogen bond. It has been found in studies that hydrogen bond can effectively modulate the electron transfer process between molecules. It can facilitate the self-assembly of molecules, endowing the material with a more ordered or crystalline structure in the solid state. This is beneficial for the charge transport across neighboring molecules in the semiconductor layers of organic field-effect transistors (OFETs) (Zhang et al., 2018a; Deng et al., 2018; Zhang et al., 2020). The charge-transfer mobility of OFETs plays a key role in ensuring their high performance; notably, however, the charge-transport mobility of the current OFETs is considerably lower than those of silicon-based field-effect transistors (Zhang et al., 2018b; Bao et al., 2020; Zou et al., 2021). Thus, the development of high-performance OFETs through structural modification to obtain hydrogen-bonded semiconductor materials with high charge-transport mobilities within individual molecules or between adjacent molecules is imperative.

Although hydrogen-bonded self-assembling materials can significantly enhance the performance of OFETs, their application has not been extensively investigated. In this minireview, small molecules and polymers of hydrogen-bonded π-conjugated materials, as well as the relationship between their chemical structures and performances in OFETs, are reviewed. In addition, this minireview provides development prospects of ideal hydrogen-bonded π-conjugated semiconductor materials with high performance.

## Hydrogen-Bonded Small Molecules

In small-molecule organic semiconductors, charge carriers need to be frequently transferred among individual molecules; therefore, the molecular crystal size and packing are crucial for efficient charge transport (Zhang et al., 2017a). Hydrogen bonding can facilitate the reorganization of the molecular packing *via* self-assembly. The charge transfer among hydrogen-bonded small molecules often results in their poor solubility in most organic solvents, owing to their strong hydrogen bonding association, which results in the formation of crosslinked netlike materials in the solid state. These materials are often difficult to utilize in the direct fabrication of OFETs *via* solution processing.

**FIGURE 1:**
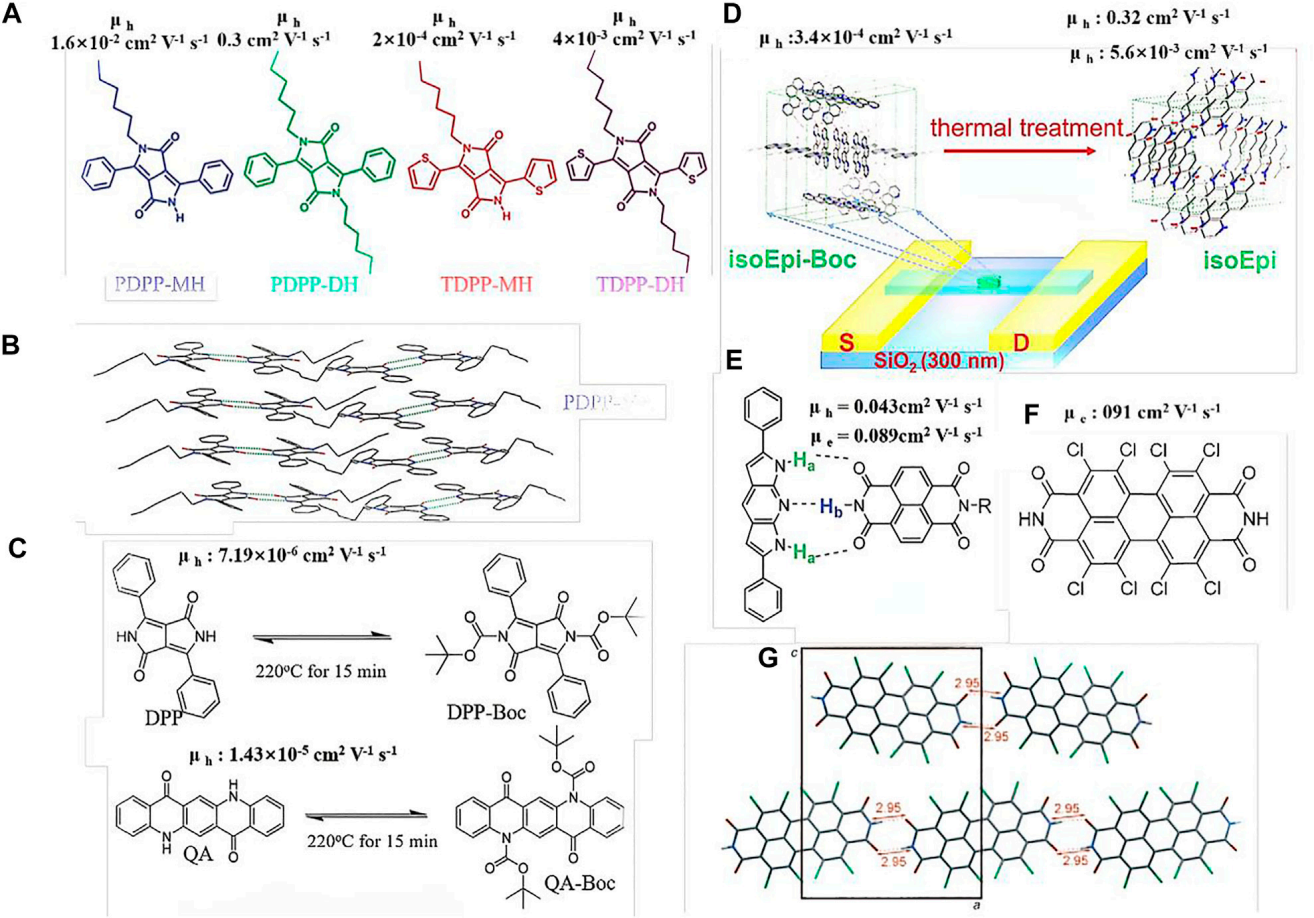
**(A,C,E,F)** Chemical structures of the small molecules; **(B,G)** crystal structures of PDPP-MH and Cl8PTCDI; **(D)** diagram of the isoEpi-Boc crystal OFET transition to hydrogen-bonded isoEpi crystal OFET.

In 2014, Patil et al*.* introduced an alkyl chain into the substituted single N position of diketopyrrolopyrrole (DPP) to obtain mono-alkylated PDPP-MH and TDPP-MH (Figure 1A) (Dhar et al., 2015). The alkyl chain not only facilitated hydrogen bonding formation, but also enhanced the material solubility. Conversely, the other NH units formed hydrogen bonds with the O = C units of neighboring molecules, resulting in dimer-like molecules (Figure 1B). PDPP-DH and TDPP-DH, which have similar chemical structures with dialkyl substituents and no hydrogen bonding, showed a hole-transfer mobility of almost two orders of magnitude higher than those of the other molecules above. Crystal analysis revealed that the mono-alkylated DPP exhibited cofacial layered structures, attributed to the intermolecular hydrogen bonding through the free amide group in the DPP core, while the dihexyl DPP-based molecules showed herring bone-packing arrangements. The present study revealed that hydrogen bonding not only affects the molecular packing, but also improves the charge-transport speed.

In addition to mono-alkylation, tert-butoxycarbonyl (t-Boc), which can be decomposed by thermal annealing or ultraviolet (UV) light exposure, has also been incorporated into small molecules *via* latent hydrogen bonding. Yanagisawa et al. incorporated t-Boc units into DPP and quinacridone (QA) to obtain t-Boc DPP and t-Boc QA, respectively (Hiroyuki et al., 2008). These two molecules exhibited good solubility in most organic solvents, permitting the fabrication of OFETs by the spin-coating technique and offering a low-cost fabrication process, rather than the expensive vacuum technology. During the thermal-annealing process at 220°C for 15 min, the t-Boc DPP and t-Boc QA molecules were converted into DPP and QA, respectively (Figure 1C). Studies on OFET devices have shown that the hole-transfer mobilities of the DPP or QA materials formed *via* t-Boc annealing are similar to those of the related DPP or QA materials formed *via* the vacuum-deposition technique. The present study demonstrated a simple and useful method to fabricate hydrogen-bonded OFETs through solution processing, which involves the introduction of a functional group that can be easily decomposed by thermal annealing, such as t-Boc, to replace the pigments with latent hydrogen bonds.

Recently, Zhang et al. reported several t-Boc-substituted conjugated pigment molecules with fused hydrogen bonds (Zhang et al., 2017b; Zhang et al., 2018a). In the present study, the authors observed that a solid-state t-Boc substituted conjugated pigment crystal could transition to a hydrogen-bonded pigment crystal *via* annealing, and the molecular packing was changed significantly. This occurred because the t-Boc units were decomposed during the thermal annealing process, while the NH units emerged (Figure 1D). Before the donor units (NH) coordinated with the acceptor units (C=O) to form hydrogen bonds, the molecules remained mobile, owing to the elevated temperature and relatively weak intermolecular interactions. Therefore, the molecules may have undergone crystallization to form hydrogen-bonded crystals. With the emergence of hydrogen bonds, the molecules were arranged to form a brick-in-wall structure with π-stacking along the crystal growth axis, leading to a significant enhancement in the charge mobility along the crystal growth direction (the hole mobility increased from 3.4 × 10^–4^ to 0.32 cm^2^ V^−1^ s^−1^, and the electron mobility increased from non-detectable to 5.6 × 10^–3^ cm^2^ V^−1^ s^−1^). The significantly improved charge-transfer mobility could be ascribed to the hydrogen bonding that not only reorganized the molecular packing, but also afforded high-density materials. It is also proved that strong hydrogen bonding between the regulatory units can induce the formation of enhanced π-π interaction (close, coplanar accumulation) and highly ordered supramolecular assembly between the conjugated units, which can better improve the migration properties of the materials. This study provides a useful strategy for preparing crystalline hydrogen-bonded small-molecule OFETs *via* solution processing.

In addition to pure conjugated materials, hydrogen bonds can be formed between two or more composite materials. In 2014, Perepichka introduced dipyrrolopyridine (DP) as a donor semiconductor capable of undergoing complementary hydrogen bonding with naphthalenediimide (NDI) acceptors (Figure 1E) (Black and Perepichka, 2014). In this system, the hydrogen bonds formed between the NH groups of the DP and the C = O or NH units of NDI were confirmed by nuclear magnetic resonance (NMR) spectroscopy and crystal analysis. Due to the hydrogen bonding, the two white or yellow materials changed into a dark–green material. The cocrystal mixture with a ratio of 1:1 (DP:NDI) exhibited relatively balanced ambipolar transport with hole and electron mobilities of 0.043 and 0.089 cm^2^ V^−1^ s^−1^, respectively, which were among the highest reported values for cocrystals at the time. This study provides a foundation for the advanced solid-state engineering of organic electronics, capitalizing on the complementary hydrogen bonding.

Most hydrogen-bonded small molecules often exhibit poor solubility in most common organic solvents, such as DPP, indigo, and octachloroperylene, due to their strong intermolecular interactions. Therefore, the OFETs based on these hydrogen-bonded molecules are often processed by the thermal vapor deposition technique. Bao et al. synthesized 1,2,5,6,7,8,11,12-tetrachloro-substituted perylene 3,4:9,10-tetracarboxylic diimides (Cl8PTCDI) and investigated their packing properties by X-ray diffraction (XRD) analysis (Figure 1F) (Li et al., 2010). The results showed that a strong hydrogen bond was formed between the two adjacent molecules with a distance of 2.95 Å, and a brick stone crystal packing arrangement was observed (Figure 1G). The crystalline Cl8PTCDI OFET showed an electron mobility as high as 0.91 cm^2^ V^−1^ s^−1^ with an air-stable operation. This high electron mobility could be due to the brick stone packing arrangement, which provides two-dimensional percolation paths for the charge-carrier transport in organic semiconductors. Recently, Geng and his team found that the addition of furan rings at 3,6-positions of DPP unit remarkably improved the solubility of the polymers (Wang et al., 2021).

## 3 Hydrogen-Bonded Polymers

Compared to that in π-conjugated small molecules, the charge transport in polymers is considerably more complicated. This is because polymers are usually semicrystalline; thus, the charge carriers need to travel across both amorphous and crystalline regions (Zhang et al., 2017a). Therefore, the charge mobility of polymers is governed not only by the crystallinity of the polymer film but also by the connections between the crystalline aggregates. Polymer is essentially between traditional semiconductor and molecular intermediates, can form a variety of nanostructures with complex energy band structures and optical properties. The hydrogen bonds with self-assembly recognition have obvious chiral inductive characteristics on the planar Π conjugated molecular skeleton. While the melting hydrogen bonds are essential for molecular rearrangement to form new solid crystals. That is to say, hydrogen bonding could facilitate the self-assembly of the molecules, affording more crystalline regions in the polymers and consequently improving the charge-carrier transfer speed.

**FIGURE 2:**
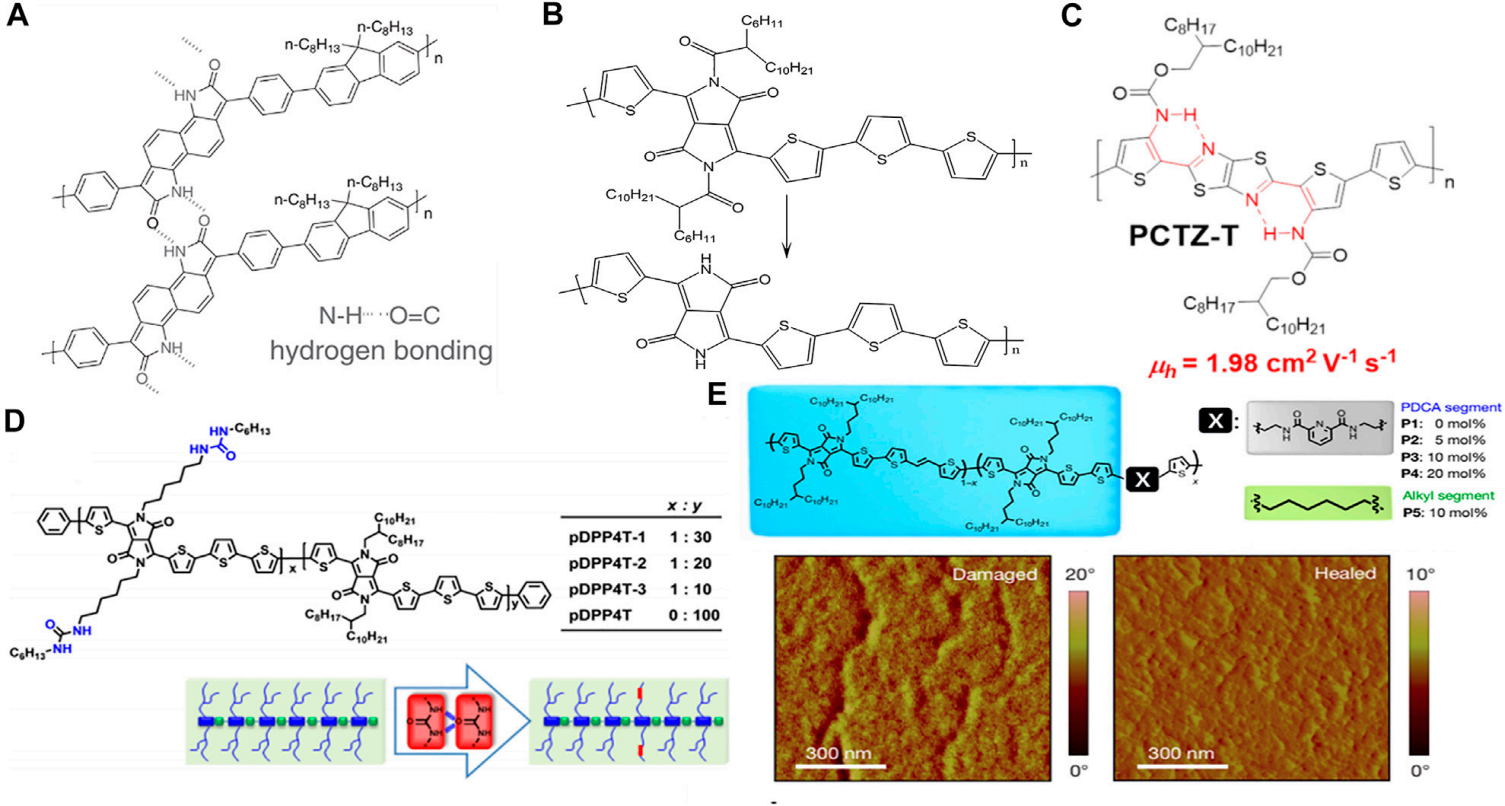
**(A)** Hydrogen-bonded cross-linked netlike NDP-based polymer; **(B)** the 2-octyldodecanoyl-substituted DPP-based polymer transition to a hydrogen-bonded DPP-based polymer; **(C)** chemical structure of polymer PCTZ-T; **(D)** chemical structures of the polymers and illustrations of the design rationale for incorporating urea groups into the side chains of conjugated polymers; **(E)** chemical structure of the DPP-based polymers, as well as the atomic force microscopy (AFM) phase image of the damaged and healed films.

Hydrogen bonds can be formed directly on the polymer backbone with conjugated units. In 2017, Zhang et al. reported two soluble π-conjugated polymers containing t-Boc-substituted benzodipyrrolidone (BDP) or naphthodipyrrolidone (NDP) units with latent hydrogen bonds on the main chain (Zhang et al., 2017b). Upon thermal annealing, the t-Boc units decomposed, forming hydrogen-bonded crosslinked netlike polymers (Figure 2A). The hydrogen-bonded polymers exhibited not only a bathochromic shift in the optical absorption and a small bandgap, but also better coplanarity and a stronger π–π interaction, compared to that of the pristine polymer. OFET studies have shown that the hydrogen-bonded polymer based on NDP affords an air-stable n-type semiconductor with an electron mobility 40 times that of its precursor polymer *via* latent hydrogen bonding. This is due to the strong aggregation and the planar polymer backbone, as confirmed by Cao et al. (Liu et al., 2015). To be solution-processable, polymer semiconductors require the incorporation of a large portion of solubilizing side chains to oppose the strong aggregation tendency of the polymer backbones in solution. To modify the t-Boc units, 2-octyldodecanoyl groups with long alkyl chains were designed and introduced into the DPP-based polymers by Li’s group (Sun et al., 2012). Similar to the t-Boc units, 2-octyldodecanoyl undergoes decomposition *via* thermal annealing while NH units emerge to form hydrogen bonds (Figure 2B). High performance organic semiconductor polymers were realized by using intramolecular resonance-assisted hydrogen bonding (RAHB). Based on this, Gao Xike’s group Liu et al. (2021) synthesized polymer PCTZ-T and PCTZ-B with Rahb interaction in the study of OFET devices, and PCTZ-T achieved an average carrier mobility of 1.98 cm^2^V^−1^s^−1^ (Figure 2C). This is the highest mobility of thiazole-containing or dythiazole-containing polymer materials in P-type OFET devices.

Apart from the polymer backbone, hydrogen bonding can also be formed with the side chain. Yao et al. introduced urea-containing alkyl chains *vs*. branching alkyl chains into DPP-based polymers to investigate the effect of hydrogen bonding on the OFET performance (Figure 2D) (Yao et al., 2016). The authors discovered that the hydrogen bonding-induced assembly significantly improved the polymer packing, typically the alky chain packing, which resulted in a thin film with thick nanofibers after thermal annealing. By increasing the ratio of hydrogen bonding from 10 to 30%, the hole-transfer mobility increased from 5.5 to 13.1 cm^2^ V^−1^ s^−1^. This study demonstrates not only the application of a urea moiety as a new functional group to design hydrogen-bonded materials, but also the incorporation of other functional moieties with hydrogen bonding into the alkyl side chains of conjugated polymers to tune the interchain interactions/packing, thereby improving the semiconductor performance.

Hydrogen bonding dissociation and association could afford a thin film semiconductor with self-healing abilities. In Oh et al. (2016) introduced non-conjugated, alkylated 2,6-pyridine dicarboxamide units into a DPP-based polymer backbone. Hydrogen bonding could be formed between the NH units and the C = O units of the neighboring molecules (Figure 2E). The linear polymer formed crosslinked polymers, owing to the hydrogen bonding, affording polymer films with hole mobilities ranging from 1.32 to 0.11 cm^2^ V^−1^ s^−1^, along the direction of the applied strain. Subsequently, the mobility recovered to 1.00 cm^2^ V^−1^ s^−1^ upon releasing the applied strain. However, the polymers with similar chemical structures and without hydrogen bonding were unable to recover upon stress release. The damaged hydrogen-bonded film could be healed by thermal annealing or solvent-vapor processing (Figure 2E). This study shows that combining hydrogen bonds into the polymer not only makes the material stretch-resistant, but also achieves efficient charge transfer.

## Conclusion and Outlook

The formation of hydrogen bonding, a strong noncovalent interaction, between neighboring molecules in the solid state results not only in molecular reassembly with more ordered and crystalline structures, but also in strong aggregations and improved π–π stacking. In addition, the hydrogen bonding could afford conjugated materials with a planar backbone. This is beneficial for the charge transfer within individual molecules and across adjacent molecules. Thus, it is possible to fabricate a good organic hydrogen-bonded conjugated semiconductor with excellent charge-transport performance. Hydrogen bonding can be formed between conjugated units, such as molecular backbones, and non-conjugated units, such as alky chains, as well as the unconjugated part of the molecular backbone. Hydrogen bonding could result in the formation of cross-linked netlike materials with poor solubility in most organic solvents; these materials cannot be utilized to build devices through solution processing. To solve this issue, t-Boc units or other functional groups can be introduced into the material structure to break the hydrogen bonding, affording soluble materials with latent hydrogen bonding. Hydrogen-bonded conjugated materials are one of the most promising semiconductor materials employed in OFETs. Thus, a series of different types of functional units with latent potential strong hydrogen bonding could be explored in the near future.
